# Pathway Analysis Incorporating Protein-Protein Interaction Networks Identified Candidate Pathways for the Seven Common Diseases

**DOI:** 10.1371/journal.pone.0162910

**Published:** 2016-09-13

**Authors:** Peng-Lin Lin, Ya-Wen Yu, Ren-Hua Chung

**Affiliations:** 1 Department of Medical Science, National Tsing Hua University, Hsin-Chu, Taiwan; 2 Division of Biostatistics and Bioinformatics, Institute of Population Health Sciences, National Health Research Institutes, Zhunan, Miaoli, Taiwan; University of Southern California, UNITED STATES

## Abstract

Pathway analysis has become popular as a secondary analysis strategy for genome-wide association studies (GWAS). Most of the current pathway analysis methods aggregate signals from the main effects of single nucleotide polymorphisms (SNPs) in genes within a pathway without considering the effects of gene-gene interactions. However, gene-gene interactions can also have critical effects on complex diseases. Protein-protein interaction (PPI) networks have been used to define gene pairs for the gene-gene interaction tests. Incorporating the PPI information to define gene pairs for interaction tests within pathways can increase the power for pathway-based association tests. We propose a pathway association test, which aggregates the interaction signals in PPI networks within a pathway, for GWAS with case-control samples. Gene size is properly considered in the test so that genes do not contribute more to the test statistic simply due to their size. Simulation studies were performed to verify that the method is a valid test and can have more power than other pathway association tests in the presence of gene-gene interactions within a pathway under different scenarios. We applied the test to the Wellcome Trust Case Control Consortium GWAS datasets for seven common diseases. The most significant pathway is the *chaperones modulate interferon signaling pathway* for Crohn’s disease (p-value = 0.0003). The pathway modulates interferon gamma, which induces the JAK/STAT pathway that is involved in Crohn’s disease. Several other pathways that have functional implications for the seven diseases were also identified. The proposed test based on gene-gene interaction signals in PPI networks can be used as a complementary tool to the current existing pathway analysis methods focusing on main effects of genes. An efficient software implementing the method is freely available at http://puppi.sourceforge.net.

## Introduction

Genome-wide association studies (GWAS) have identified thousands of single nucleotide polymorphisms (SNPs) significantly associated with complex diseases [1], such as Crohn’s disease and type 2 diabetes [2, 3]. Traditional GWAS analyses focused on testing the associations between individual SNPs and the disease. However, for SNPs with modest effects, GWAS has low power to detect such SNPs because of the high multiple testing correction burden resulting from the large number of tests (e.g., 1 million tests) typically performed in GWAS. Moreover, the power for GWAS can be limited by the sample size for a study. For example, more than 5,000 cases and the same number of controls are required for a GWAS to achieve power > 80% at the genome-wide significance level for SNPs with effect sizes between 1.3 and 1.5 [4].

Pathway analysis has become popular as a secondary analysis strategy for GWAS data. Pathway analysis hypothesizes that SNPs in genes in the same pathway have a joint effect on the disease. One of the advantages of pathway analysis is that the statistical power for identifying disease susceptibility genes can be increased by the joint modeling of the effects of SNPs. Another advantage is that the results can provide biologically meaningful insights into the complex disease mechanism. Furthermore, multiple testing correction burden can be reduced in pathway analysis by testing hundreds or thousands of pathways instead of testing hundreds of thousands or a million of SNPs.

Current statistical methods for pathway analysis using GWAS data can be divided into two categories (i.e., competitive tests and self-contained tests) based on their null hypotheses [5]. The competitive tests compare the distribution of statistics for genes within a given pathway to the distribution of statistics for other genes across the genome. Some examples for this type of methods include Wang’s method [6] extended from the gene-set enrichment analysis (GSEA) [7], ALIGATOR [8], and Pathway-PDT [9]. In contrast, the self-contained tests compare the distribution of statistics for genes within the given pathway to the statistics for the same genes under the null. Methods such as the set-based test in PLINK [10], GRASS [11] and OPTPDT [12] are in this category. The self-contained tests can be more powerful than the competitive tests, due to the more restrictive null hypothesis for the tests than the null for the competitive tests [13]. The statistics for the aforementioned methods were constructed based on the individual effects of SNPs. However, gene-gene interaction effect, which is referred to as the departure from a combination of individual marginal effects [14], can also play a role in complex disease etiology [15]. Incorporating gene-gene interactions in pathway analysis thus becomes important.

Testing gene-gene interactions in GWAS is challenging because a very large number of interaction pairs needs to be examined, which is computationally expensive and results in a high multiple testing correction burden. For example, there are hundreds of billions of possible SNP pairs for a GWAS with 1 million SNPs. With the increased biological knowledge of protein-protein interactions (PPI), several public PPI databases are available, such as STRING [16] and BioGRID [17]. PPI has been defined as functional epistasis, while gene-gene interaction discussed here has been defined as statistical epistasis [18]. PPI has been found to be associated with complex diseases [19]. Moreover, experimental results from the yeast studies have suggested a connection between functional and genetic epistasis [18]. For example, in the study by St. Onge et al. [20], which examined the genetic interactions influencing the resistance of yeast to the DNA-damaging agent methyl methanesulphonate, nine of the ten genetic interactions that they identified encoded or were predicted to encode physical protein interactions. Moreover, a genome-wide construction of a genetic interaction map for the budding yeast has also identified 10–20% overlap between genetic interactions and protein-protein interactions [21]. PPI can also be used in human disease studies as an informative prior for searching disease genes [22, 23]. For example, some studies have performed gene-gene interaction analysis for GWAS incorporating PPI information by only testing SNPs at genes in the same PPI network [24–27], and significant SNP interaction pairs have been identified for complex traits such as Crohn’s disease, bipolar disorder, hypertension, rheumatoid arthritis, and high-density lipoprotein cholesterol levels.

Several network-based tests have been proposed to identify gene networks associated with the disease also based on PPI networks, without using prior knowledge of pathway definitions [28]. For example, a dense module searching (DMS) method was developed to identify genes in a subnetwork with low p-values compared to background genes from the entire PPI networks, while the p-value for each gene is the minimum association p-value for SNPs within the gene [29]. In NIMMI [30], the Google PageRank algorithm was used to calculate weights for genes in the same PPI networks. The weights, along with association p-values for genes, were used to calculate weighted gene scores. Genes with high gene scores were then analyzed for functional relationship using DAVID [31]. These methods, however, still used association p-values from the tests for the main effects of SNPs without specifically considering statistical evidence from gene-gene interactions.

As multiple gene-gene interactions can occur within a pathway [32], combining gene-gene interaction signals within the pathway can increase the power to detect the effects. Previously we developed the Pathway analysis method Using Protein-Protein Interaction network for case-control data (PUPPI) [33]. PUPPI only considers pairs of genes in the PPI network within a pathway for the interaction analysis, and an overall statistic for the pathway is calculated. The main difference between the PUPPI and existing pathway or network-based methods is that the PUPPI statistic is constructed based on gene-gene interaction test statistics, instead of the test statistics for main effects. Therefore, the PUPPI is able to identify pure epistasis (i.e., interaction without main effects) within a pathway. Here, we performed a more comprehensive simulation study to evaluate the type I error rates for the PUPPI and compare the power for the PUPPI with other methods. We then applied the PUPPI to the Wellcome Trust Case Control Consortium (WTCCC) GWAS datasets [34] for seven common diseases, and identified several significant pathways that have implications in the diseases.

## Materials and Methods

### The PLINK interaction statistic

We first review the PLINK interaction statistic (i.e., the—fast-epistasis option in PLINK) as the PUPPI was developed based on the statistic. Two 2 by 2 allele tables, collapsed from two 3 by 3 genotype tables, are created separately for cases and controls. For example, assume we categorize all cases into a 3 by 3 table based on their genotypes at two SNPs, where one SNP has genotypes *AA*, *Aa*, and *aa*, and the other SNP has genotypes *BB*, *Bb*, and *bb*, as shown in Table 1. A 2 by 2 table for alleles can be subsequently constructed by collapsing the 3 by 3 table, as shown in Table 2, where each cell count is the observed number of alleles in the sample. An odds ratio is calculated based on each of the 2 by 2 tables. The interaction statistic is then calculated as:

$$Z=\frac{log({OR}_{case})-log({OR}_{control})}{\sqrt{Var[log({OR}_{case})-log({OR}_{control})]}}$$ (1)

where *OR*_*case*_ and *OR*_*control*_ are the odds ratios calculated based on the 2 by 2 tables for cases and controls, respectively. Assuming that the two SNPs are in Hardy-Weinberg Equilibrium (HWE) and linkage equilibrium (LE), the statistic follows a standard normal distribution under the null hypothesis of no gene-gene interaction for the two SNPs.

**Table 1:** Genotype table for two SNPs. Each cell count is the number of individuals with the specific genotype.

	*BB*	*Bb*	*bb*
*AA*	*a*	*b*	*c*
*Aa*	*d*	*e*	*f*
*aa*	*g*	*h*	*i*

**Table 2:** Allele table for two SNPs. Each cell count is the number of alleles in the sample collapsed from Table 1.

	*B*	*b*
*A*	*4a+2b+2d+e*	*4c+2b+2f+e*
*A*	*4g+2h+2d+e*	*4i+2h+2f+e*

### The PUPPI algorithm

The PUPPI algorithm was previously described in our conference paper [33]. Here, we provide more details in the algorithm. Assume ψ is a set of pairs of genes with known protein-protein interactions within pathways, and the same two genes are either on different chromosomes or more than *k* MB apart on the same chromosome. Because the PLINK interaction statistic assumes there is no LD between SNPs tested for interaction, we consider pairs of genes that are not linked if they are on the same chromosome. The value of *k* was set as 1 in our real data analysis. For each pair of genes in ψ, the PLINK interaction statistics are calculated for all possible pairs of SNPs between the two genes. Then the maximum statistic *M* from the statistics for all pairs of SNPs between the two genes is selected.

A gene pair with large gene sizes can generate larger *M* than a gene pair with small gene sizes because *M* was selected from a larger set of interaction statistics for the pair of large genes. We therefore adjust *M* by gene size so that large genes do not contribute more to the pathway statistic simply due to their size. The effective numbers [35] are used to adjust for gene size in the statistics. Effective numbers are estimated based on the principal component analysis (PCA) approach. The effective number estimates the number of independent SNPs for a set of SNPs. Assume that the effective number is *S*_*eff*_ estimated from a set of *S* SNPs. In Babron et al. [36], an effective number of all pairwise SNPs in *S* was calculated as *S*_*eff*_ (*S*_*eff*_ -1)/2, which is the number of all pairwise combinations from the set of *S*_*eff*_ elements. Their simulation results suggest that the number slightly overestimated the real effective number of independent SNP pairs. Similarly, assume that *m* and *n* are the effective numbers for the SNPs in the gene pair, where SNPs between the gene pair are independent, then *m*×*n* estimates the number of independent tests between the two genes. The adjusted statistic *M*′ for *M* is calculated as:

$$M^{\prime }=\left\{ \begin{array}{l} \Phi^{-1}(1-(pvalue\;for\;M)\times m\times n)\;if\;(pvalue\;for\;M)\times m\times n<l\\ \;0\;otherwise \end{array}\right.$$ (2)

where Φ(*x*) is the cumulative distribution function for the random variable *x* following a chi-square distribution with 1 degree of freedom. In Eq 2, the Bonferroni correction is first applied to the p-value for *M*, calculated based on a standard normal distribution. The adjusted statistic *M*′ is the statistic corresponding to the p-value with the Bonferroni correction if the adjusted p-value is less than *l*. The adjusted statistic *M*′ is set as 0 if the adjusted p-value is ≥ *l*.

The PUPPI statistic *X* for pathway *i* is the sum of the adjusted statistics *M’* for gene pairs in the pathway. A permutation procedure, which permutes the case-control affection status, was used to approximate the distribution of *X* and calculate the p-value. The null hypothesis for the PUPPI is that there are no interaction effects between genes on the disease within the pathway. As the PUPPI compares the test statistic to the same test statistics for the same genes under the null, the PUPPI is also a self-contained test. The PUPPI algorithm is summarized in the following steps:

1. For each gene in ψ, calculate the effective number for SNPs in the gene.
2. The PLINK interaction statistics are calculated for all pairs of SNPs between each pair of genes in ψ.
3. The maximum statistic *M* from the statistics for all pairs of SNPs between two genes in ψ is selected, and the adjusted statistic *M*′ is calculated based on Eq 2.
4. The PUPPI statistic *X* for pathway *i* is the sum of *M*′ for gene pairs in the pathway.
5. Perform permutations for *K* times. For each permutation, steps 2–4 are repeated and a permuted PUPPI statistic *M*_*p*_′ is calculated. The p-value for pathway *i* is calculated as (# of *M*_*p*_′ ≥ *M*′)/*K*.

### Simulations

We used computer simulations to evaluate the type I error rates for the PUPPI, and to compare the power of the PUPPI with other methods under different scenarios. SeqSIMLA2 [37] was used to generate simulated replicates of cases and controls. We first used HapGen2 [38] to simulate 10,000 haplotypes with similar frequencies and LD structures to those in the Utah Residents (CEPH) with Northern and Western European Ancestry (CEU) samples from the HapMap3 project. Haplotypes in genes in the Glycolysis/Gluconeogenesis pathway (hsa00010) and the Pentose phosphate pathway (hsa00030) defined in KEGG [39] were simulated. The 10,000 haplotypes were then adopted by SeqSIMLA2 to simulate unrelated cases and controls. To generate SNP sets similar to a GWAS platform, SNPs that are on the Affymetrix 6.0 array and with minor allele frequencies (MAF) > 1% were extracted from the simulated replicates. For a pathway, SNPs in genes that are not in the PPI networks were excluded. The PPI networks were downloaded from the STRING database [16], which will be discussed in more detail in the next section. A total of 366 SNPs in 44 genes and 138 SNPs in 15 genes were analyzed for the hsa00010 and hsa00030 pathways, respectively. The parameter *l* in Eq 2 was set as 0.05 in all simulation studies and real data analysis.

For type I error simulations, we simulated three different sample sizes, including 500 cases and 500 controls, 1,000 cases and 1,000 controls, and 2,000 cases and 3,000 controls, for the two pathways. For power studies, we simulated a scenario where there were both main effects and interaction effects for the disease SNPs (Scen1). We also simulated another scenario where there were only interaction effects (i.e., pure epistasis) for the disease SNPs (Scen2). For Scen1, the four epistasis models (Models 1–4) used in Wan et al. [40] were adopted in our simulations. The models included a model used to describe handedness and the color of swine (Model 1), an exclusive OR model (Model 2), a multiplicative model (Model 3), and a classical epistasis model (Model 4). We considered disease heritability of 0.01 and 0.025 for the four models, which resulted in a total of 8 scenarios. The penetrance functions for the 8 scenarios are shown in S1 File. For Scen2, six of the pure epistasis models without main effects in Wan et al. [40] (i.e., Models epi 41, 42, 51, 52, 61, and 62) were used. Penetrance functions for the 6 models, corresponding to heritability of 0.05, 0.025, and 0.01, were provided in the Supplementary materials in Wan et al. [40]. To simulate multiple gene-gene interactions within a pathway, we selected three pairs of SNPs from three different pairs of genes in the hsa00030 pathway as the disease SNPs. The MAF for the disease SNPs were close to 0.2. We assumed 50%, 25%, and 25% of cases were caused by the interactions from each of the three pairs of disease SNPs, respectively, in each simulated replicate, which resulted in samples with genetic heterogeneity.

We compared the power of the PUPPI with three other self-contained tests, the PLINK set-based test, HYST [41], and SKAT [42]. The pathway statistics for the PLINK set-based test and HYST were constructed based on the statistics for testing the main effects of individual SNPs. The PLINK set-based test considered SNPs in genes within a pathway as a whole set, without specifically modeling the relationship between SNPs and genes. HYST considered LD blocks as test units and aggregated p-values for LD blocks within a pathway for the test. Both tests have been shown to be powerful tests compared to other existing pathway association tests [11, 41]. SKAT is a kernel-based testing approach, which constructs a variance-component score test statistic for SNP-set analysis. In contrast to the PLINK set-based test and HYST that consider only main effects, the “2wayIX” kernel was specified in SKAT, which accounted for both main effects and interaction effects.

### Pathway analyses for the WTCCC datasets

We downloaded the pathway definitions based on the KEGG [39], REACTOME [43], and Biocarta (http://www.biocarta.com) databases from the Molecular Signatures Database (MSigDB) on the GSEA website (http://www.broadinstitute.org/gsea). We downloaded the PPI information from the STRING database [16]. Each pair of PPIs in STRING has a confidence score, which was calculated based on the combination of probabilities of PPIs from different sources, such as the KEGG database, public literatures, and functional genomics data [44]. A PPI with a score > 0.7 was considered as high confidence in STRING. We extracted PPI pairs with scores > 0.8 in STRING for the analysis to ensure a high quality set of PPIs. We downloaded the hg18 gene annotations from the UCSC genome browser website [45]. We applied the PUPPI to the WTCCC GWAS datasets for the pathway analyses. The datasets consisted of about 3,000 shared controls and 2,000 cases for each of the seven diseases, including bipolar disorder (BD), coronary artery disease (CAD), Crohn’s disease (CD), hypertension (HT), rheumatoid arthritis (RA), type 1 diabetes (T1D), and type 2 diabetes (T2D). The same quality control (QC) procedures as those used in the WTCCC studies were performed on the datasets. The analysis in the present study was approved by the Institutional Review Board of the National Health Research Institutes in Taiwan (IRB protocol # EC1020503-E), and written informed consent was obtained from all subjects.

## Results and Discussion

### Simulation results

Table 3 shows the type I error rates for the PUPPI under different scenarios. The PUPPI maintained proper type I error rates with different samples sizes and different sizes of pathways at the 0.05 and 0.01 significance levels. All of the 95% confidence intervals (CI) shown in the Table contained the expected values.

**Table 3:** Type I error rates for PUPPI with different sample sizes and pathways.

			Type I error (95% CI)	
Pathway	Number of SNPs	Sample size	α = 0.05	α = 0.01
hsa00010	366	2000 cases and 3000 controls	0.0506 (0.0445,0.0566)	0.0078 (0.0050,0.0105)
hsa00010	366	1000 cases and 1000 controls	0.0490 (0.0430,0.0550)	0.0128 (0.0100,0.0156)
hsa00010	366	500 cases and 500 controls	0.0512 (0.0452,0.0572)	0.0126 (0.0098,0.0154)
hsa00030	138	2000 cases and 3000 controls	0.0526 (0.0465,0.0586)	0.0108 (0.0080,0.0136)
hsa00030	138	1000 cases and 1000 controls	0.0486 (0.04250.0546)	0.0128 (0.0100,0.0156)
hsa00030	138	500 cases and 500 controls	0.0524 (0.0464,0.0584)	0.0120 (0.0092,0.0148)

**Fig 1:**
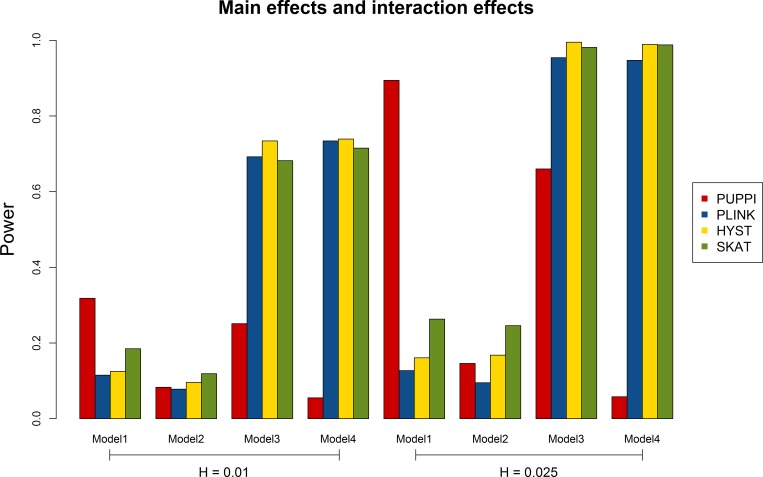
Power comparison for the PUPPI with the PLINK set-based test, HYST, and SKAT at the significance levels (alpha) of 0.05 and 0.01 under models with both main effects and interaction effects.

Fig 1 shows the power comparison in the presence of both main effects and interaction effects for 2,000 cases and 3,000 controls. Different power patterns were observed for different models. Under Model 1 that was used to describe some real traits, the PUPPI can have significantly higher power than the other tests with either heritability (H) of 0.01 or 0.025. For the XOR model (Model 2), SKAT had the highest power compared to the other three tests. PUPPI and the PLINK set-based test had similar power, while HYST had a little more power compared to them. HYST, the PLINK set-based test, and SKAT can have significantly higher power in the multiplicative model (Model 3) and the classical epistasis model (Model 4) than the PUPPI. Moreover, HYST had more power than the PLINK set-based test in all of the models.

**Fig 2:**
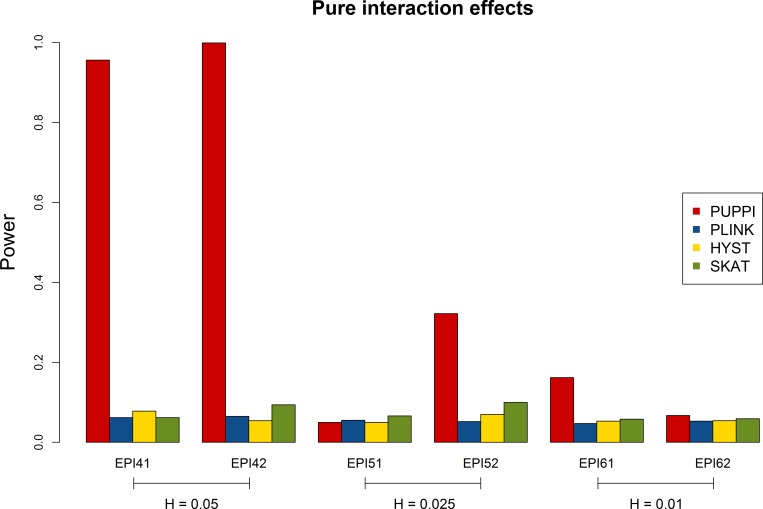
Power comparison for the PUPPI with the PLINK set-based test, HYST, and SKAT at the significance levels (alpha) of 0.05 and 0.01 under models with only interaction effects.

Fig 2 shows the power comparison under the pure epistasis models also for 2,000 cases and 3,000 controls. The PLINK set-based test and HYST showed power close to the 0.05 significance level across all models. This is as expected because there were no main effects for the disease SNPs, which was under the null hypothesis for the two tests. Although interaction effects were considered, SKAT only had somewhat higher power than 0.05 under most of the models. In contrast, the PUPPI can have high power in some models, such as EPI41 and EPI42. The power results demonstrated the advantage of using the PUPPI for detecting pure epistasis within pathways, which cannot be identified by pathway methods based on testing for the main effects of SNPs.

### Overall results for the WTCCC pathway analyses

A total of 1,078 pathways were downloaded from the GSEA website. There were 423,220 PPI pairs with scores > 0.8 in the STRING database. After QC, the WTCCC datasets consisted of 2,938 shared controls, 1,868 BD cases, 1,926 CAD cases, 1,748 CD cases, 1,952 HT cases, 1,860 RA cases, 1,963 T1D cases, and 1,924 T2D cases. There were 457,710 SNPs left for the analysis. After adjusting for multiple testing based on the familywise error rates (FWERs) or false discovery rates (FDRs) using the methods described in Wang et al. [6], none of the pathways were significant. Therefore, we defined pathways with the PUPPI p-values < 0.05 as significant pathways and focused on functional interpretations for the significant pathways. The most significant pathways identified by the PUPPI which have functional implications in the seven diseases are shown in Table 4. A majority of the pathways shown in Table 4 are actually the most significant pathways in the individual disease analyses. The significant pathways with p-values<0.05 for each disease are shown in S2 File. The significant pathway with functional implications for each disease is discussed as follows.

**Table 4:** The most significant pathways that have functional implications for the seven diseases.

Disease	Database	Pathway names	P-value	Rank^1^
BD	REACTOME	Metal ion SLC transporters	0.0030	1
CAD	BIOCARTA	Acetylation and deacetylation of RelA in the nucleus	0.0036	1
CD	BIOCARTA	Chaperones modulate interferon signaling pathway	0.0003	1
HT	REACTOME	Mitochondrial protein import	0.0014	3
RA	KEGG	Complement and coagulation cascades	0.0072	2
T1D	BIOCARTA	IL-7 signal transduction	0.0044	1
T2D	REACTOME	Signaling by FGFR3 mutants	0.0008	1

^1^The rank of the pathways based on the p-values in the individual disease analysis

### BD

For BD, the significant pathway, *Metal ion SLC transporters*, contains the pathways which transport ions such as Cu^2+^, Fe^2+^, Fe^3+^, Mg^2+^, Mn^2+^, Zn^2+^, etc. The solute carrier (SLC) is a group of membrane transport proteins. The genes solute carrier family 30 member 3, member 6 and member 7 (SLC30A3, SLC30A6, and SLC30A7), and solute carrier family 39 member 6 (SLC39A6) encoding zinc transporters are expressed in the brain [46–49]. The essential metal ion zinc can induce oxidative damage in the brain and the strict regulation of zinc can protect the brain from injury [50].

### CAD

The functions for the significant pathway for CAD are acetylation and deacetylation of RelA in the nucleus. RelA (p65) is a member of the NFκB family, consisting of transcription factors regulating mainly the immune response, and having some functions in heart. RelA has been implicated in cardiac remodeling [51], which is the expansion and shrinkage of coronary vessels. RelA can be acetylated by the CREB-binding protein (CBP) and p300 protein in this pathway. In fact, CBP and p300 have significant gene-gene interaction (*M’* = 7.26) in the PUPPI test. Moreover, the interaction between RelA and CBP/p300 is modulated by protein kinase A (PKA), which can phosphorylate RelA. Such reaction may induce cardiac remodeling, an important process in the development of coronary artery disease [52].

### CD

The *chaperone modulate interferon signaling* pathway for CD is the most significant pathway in the overall analyses. The protein hTid-1 is a chaperone that modulates interferon signaling and can also repress NFκB [53]. Persistent inhibition of NFκB leads to inappropriate immune cell development [54]. Moreover, interferon gamma is a member of the macrophage activating factor, which is a lymphokine that can activate macrophages. In Crohn’s disease patients, the defective macrophage function may play a role [55]. More interestingly, one of the actions of interferon in this pathway is to induce mitochondria to activate apoptosis, which has been found to increase in Crohn’s disease patients [56]. This pathway can also induce the downstream JAK/STAT pathway, which can regulate certain immune systems.

Table 5 shows the significant gene pairs with *M’* values not equal to 0 in the chaperone pathway. The most significant gene pair, interferon gamma receptor 1 (IFNGR1) and interferon gamma receptor 2 (IFNGR2), are the receptors of interferon gamma. A cross-linking experiment has shown that IFNGR2 is associated with interferon gamma only when the IFNGR1 chain is present [57]. If interferon gamma fails to bind to IFGR1 and IFGR2, it cannot trigger many functions of the pathway. The three genes, v-rel avian reticuloendotheliosis viral oncogene homolog A (RELA), nuclear factor of kappa light polypeptide gene enhancer in B-cells 1 (NFKB1), and inhibitor of kappa light polypeptide gene enhancer in B-cells, kinase beta (IKBKB), in other significant gene pairs in Table 5 have been identified to have signal-induced protein interactions in the *in vivo* screen tests [58]. These three genes are all highly associated with inflammatory response [58]. Therefore, interactions among the three genes can also have effects on the disease.

**Table 5:** Significant gene pairs in the chaperone pathway for CD.

Significant gene pair	*M’*
(IFNGR2)(IFNGR1)	6.750239
(IKBKB)(NFKB1)	5.65706
(RELA)(NFKB1)	5.199413
(TNF)(IFNG)	5.131393
(IKBKB)(TP53)	4.686214

### HT

The function of the significant pathway for HT is related to the import of mitochondrial protein. Hypertension is associated with the elevation level of reactive oxygen species (ROS) [59], and the reactions of ROS take place mainly in mitochondria. Mitochondria dysfunction may cause hypertension, and then generate excessive ROS to damage mitochondrial DNA, which causes a vicious cycle in the hypertension state.

### RA

The *Complement and coagulation cascades* pathway has three stages: the complement cascade, the Kallikrein-Kinin cascade, and the coagulation cascade. For the complement cascade, the complement activation can recruit the inflammatory and immunocompetent cells to kill the pathogens. This cascade has three pathways: the alternative pathway, the lectin pathway and the classical pathway. All three pathways are related to the complement system, which helps the antibodies and phagocytic cells to remove the pathogen from the body. In the lectin pathway, the gene pair mannose-binding lectin 2 (MBL2)-mannan-binding lectin serine peptidase 1 (MASP1) has a significant interaction in the PUPPI test (*M’* = 10.42). The gene MBL2 encodes mannose-binding lectin, which can recognize microorganisms. MASP1 can play a role as an enzyme to interact with MBL2 to activate the lectin pathway [60]. The second cascade is the kallikrein-kinin system. When this system is triggered, it will release vasoactive kinin. Kallikrein–kinin proteins play an important role in the pathophysiology of rheumatoid arthritis [61]. In the coagulation cascade, coagulation factor II (thrombin) can activate the coagulation factor II receptor (also known as the protease-activated receptor, PAR). PAR can regulate inflammation. Thus activation of coagulation will enhance PCR and then promote the inflammation [62].

### T1D

The IL-7 signal transduction pathway can lead to immune response. Interleukin-7 (IL-7) is a cytokine which can trigger the immune system to develop B-cells and T-cells. In the etiology of type 1 diabetes, IL-7 is believed to be involved in the infiltration of the effector T-cells into pancreatic beta cells [63]. Two studies suggested that blockage of the IL-7 receptor can help to treat type 1 diabetes in non-obese mice [64, 65]. Thus, the pathway is a candidate pathway for type 1 diabetes.

### T2D

The *Signaling by FGFR3 mutants* pathway for T2D is also promising. T2D patients have dysfunctional β-cells in the islets [66]. Fibroblast growth factor receptor 3 (FGFR3) signaling can inhibit the expansion of pancreatic epithelial cells. It has been suggested that some of the pancreatic epithelial cells (the precise type is unclear) can differentiate β-cells [67]. FGFR3 is also involved in the regulation of pancreatic growth when the mature islet cells emerge [68].

Some pathways, while not the most significant for a given disease in our analyses for the WTCCC data, are nonetheless also functionally promising for that disease. For HT, the fourth (the *downregulation of TGF-β receptor signaling* pathway with p-value = 0.0014) and fifth (the *TGF-β receptor signaling activates Smad* pathway with p-value = 0.0018) significant pathways are both related to TGFβ signaling. In fact, the fourth significant pathway is a part of the TGF-β receptor signaling, which activates the Smad pathway. TGFβ is expressed more in patients with hypertension than in the normal controls [69]. The TGFβ/Smad signaling pathway can induce vascular fibrosis, which is a pivotal aspect of vascular remodeling in hypertension [70, 71]. For RA, the *phosphoinositides and their downstream targets* pathway is the third significant pathway, with p-value = 0.0088. This pathway shows the downstream target of phosphoinositides, which can be added to a phosphate molecule on the 3 position of inositol by phosphoinositide 3-kinase (PI3K), which is a subfamily of lipid kinase. The target downstream of PI3K can control many cell functions, such as proliferation, migration, and survival [72, 73]. PI3Kγ and PI3Kδ can trigger several immune responses, and have crucial roles in the progress in RA [73].

The pathways we found based on gene-gene interaction tests are different from those found by the single-locus strategy, which focused on testing main effects [74]. However, some of them have similar functions. For example, the JAK/STAT pathway was previously found to be associated with Crohn’s disease based on signals from single-locus SNPs [5]. Interestingly, hTid-1 (in the chaperone pathway identified in our analysis) modulates interferon gamma, which induces the JAK/STAT pathway. Therefore, both pathways may be involved in the etiology of Crohn’s disease.

## Conclusions

We performed simulation studies to verify that the PUPPI has correct type I errors for pathways with different numbers of genes and for different sample sizes. As PPI information is independent from the statistical tests, it is important to note that using PPI information in the PUPPI does not bias the test statistics. The power simulation results suggested that the PUPPI can have higher or comparable power to that of PLINK, HYST, and SKAT in some models when there were both main effects and interaction effects. Moreover, for the pure epistasis models, the PUPPI can have high power while tests based on main effects would not have power to identify the effects. Therefore, the major advantage of the PUPPI over other pathway analysis methods based on testing for main effects is that pure epistasis within a pathway can be identified. The PUPPI can be used as a complementary test to the tests based on main effects. That is, PLINK and HYST can be used to identify pathways containing SNPs with main effects on the disease, and the PUPPI can be further used to identify pathways with gene-gene interaction effects. Furthermore, it is possible to incorporate main effects of SNPs in the PUPPI algorithm. For example, statistics for main effects can be calculated for individual SNPs in Step 2 in the PUPPI algorithm, and a statistic combining statistics for gene-gene interaction and main effects can be calculated in Step 3. Further research will be required to evaluate the statistical properties for the method.

The Bonferroni correction in Eq 2 can be a conservative correction for the p-value. A similar procedure to the modified Simes procedure [75] may be adopted in the PUPPI as an alternative approach to correcting for the p-value. However, using the procedure will require the calculations of effective numbers for subsets of SNPs, which will increase the computational burden in the PUPPI. Moreover, the modified Simes procedure was designed for p-values from individual SNPs. More research will be required to investigate the extension of the procedure to the gene-gene interaction p-values.

The application of the PUPPI to the WTCCC datasets identified several promising pathways. However, their p-values were not significant after correcting for multiple testing. Some methods or algorithms to improve the power for pathway association tests for GWAS have been discussed extensively in the literature [5, 76, 77]. For example, the identification of an optimal p-value threshold *l* to calculate the PUPPI statistic *M*′ may increase the power. This can be achieved by algorithms using multiple p-value thresholds [12]. Moreover, with prior biological knowledge, more weights can be assigned to damaging SNPs in the pathway statistic. Furthermore, increasing the SNP density by imputing untyped SNPs based on a reference panel such as the 1000 Genomes Project [78] data may also increase the power for the analysis [8].

In conclusion, our analyses demonstrate that pathway analysis using gene-gene interactions can be useful for identifying pathways associated with the disease. The analysis can complement the pathway analysis using only signals from single-locus SNPs. The PUPPI is implemented with C++ incorporating POSIX Threads (Pthreads) to parallelize the code. The program can be downloaded for free from the website: http://puppi.sourceforge.net.

## Supporting Information

S1 File Penetrance functions for the 4 models with both main effects and interaction effects. (DOCX)

S2 File The PUPPI analysis results with p-values<0.05 for the seven diseases. (XLSX)
