# Supplementary material for: Pathway Analysis Incorporating Protein-Protein Interaction Networks Identified Candidate Pathways for the Seven Common Diseases
Source: PLoS One. 2016 Sep 13;11(9):e0162910. doi: 10.1371/journal.pone.0162910 (PMC5021324; doi:10.1371/journal.pone.0162910)
Supplement: S1 File — (DOCX) [file pone.0162910.s001.docx]

**Model 1:**

Heritability=0.01

|  | AA | Aa | aa |
| --- | --- | --- | --- |
| BB | 0.091 | 0.148 | 0.148 |
| Bb | 0.148 | 0.091 | 0.091 |
| bb | 0.148 | 0.091 | 0.091 |

Heritability=0.025

|  | AA | Aa | aa |
| --- | --- | --- | --- |
| BB | 0.091 | 0.178 | 0.178 |
| Bb | 0.178 | 0.091 | 0.091 |
| bb | 0.178 | 0.091 | 0.091 |

**Model 2:**

Heritability=0.01

|  | AA | Aa | aa |
| --- | --- | --- | --- |
| BB | 0.091 | 0.148 | 0.091 |
| Bb | 0.148 | 0.091 | 0.148 |
| bb | 0.091 | 0.148 | 0.091 |

Heritability=0.025

|  | AA | Aa | aa |
| --- | --- | --- | --- |
| BB | 0.091 | 0.181 | 0.091 |
| Bb | 0.181 | 0.091 | 0.181 |
| bb | 0.091 | 0.181 | 0.091 |

**Model 3:**

Heritability=0.01

|  | AA | Aa | aa |
| --- | --- | --- | --- |
| BB | 0.091 | 0.091 | 0.091 |
| Bb | 0.091 | 0.148 | 0.234 |
| bb | 0.091 | 0.234 | 0.484 |

Heritability=0.025

|  | AA | Aa | aa |
| --- | --- | --- | --- |
| BB | 0.091 | 0.091 | 0.091 |
| Bb | 0.091 | 0.177 | 0.316 |
| bb | 0.091 | 0.316 | 0.681 |

**Model 4:**

Heritability=0.01

|  | AA | Aa | aa |
| --- | --- | --- | --- |
| BB | 0.091 | 0.091 | 0.180 |
| Bb | 0.091 | 0.180 | 0.091 |
| bb | 0.180 | 0.091 | 0.091 |

Heritability=0.025

|  | AA | Aa | aa |
| --- | --- | --- | --- |
| BB | 0.091 | 0.091 | 0.227 |
| Bb | 0.091 | 0.227 | 0.091 |
| bb | 0.227 | 0.091 | 0.091 |
